# Associations of increases in public transport use with physical activity and adiposity in older adults

**DOI:** 10.1186/s12966-018-0660-x

**Published:** 2018-04-02

**Authors:** Anthony A. Laverty, Elizabeth Webb, Eszter P. Vamos, Christopher Millett

**Affiliations:** 1 Public Health Policy Evaluation Unit, School of Public Health, Imperial College London, London, UK; 2 ESRC International Centre for Lifecourse Studies in Society and Health, London, UK

**Keywords:** Physical activity, Public transport, Active transport, Older adults

## Abstract

**Background:**

We investigated predictors of two increases in older people’s public transport use: initiating public transport use among non-users; and increasing public transport use amongst users. We also investigated associations of these changes with physical activity, Body Mass Index (BMI) and waist circumference.

**Methods:**

Data come from the 2008 and 2012 English Longitudinal Study of Ageing (ELSA). Logistic regression assessed predictors of increases in public transport use among adults aged ≥50 years. Gender-stratified logistic and linear models assessed associations of increases in public transport use with changes in physical activity and adiposity.

**Results:**

Those becoming eligible for a free older person’s bus pass were more likely to both initiate and increase public transport use (e.g. for initiating public transport use Adjusted Odds Ratio (AORs) 1.77, 95% Confidence Interval 1.35; 2.33). Retiring from paid work was also associated with both initiating and increasing public transport use e.g. AOR 1.57 (1.29; 1.91) for initiating use.

Women who increased public transport use had mean BMI 2.03 kg/m^2^ lower (− 2.84, − 1.21) at follow up than those who did not, although this was attenuated after adjusting for BMI at baseline (− 0.40 kg/m^2^, − 0.82, 0.01). After adjustment for baseline physical activity those initiating public transport use were more likely to undertake at least some physical activity in 2012 (e.g. AOR for women 1.67, 1.03; 2.72).

**Conclusions:**

Both initiating and increasing public transport use were associated with increased physical activity and may be associated with lower adiposity among women. These findings strengthen the case for considering public transport provision as an effective means of promoting healthier ageing.

## Background

Increasing population levels of physical activity through the promotion of physically active forms of travel is now recognised as a potential mechanism to combat obesity levels and non-communicable diseases (NCDs) [1, 2]. Meta-analyses have linked active transport to increases in physical activity and reductions in adiposity as well as cardiovascular disease risk [3, 4]. The majority of research and policies however, have focused on walking and cycling as these are considered to be the most physically active travel modes [5]. More recent research has investigated whether public transport is also associated with the benefits conferred by walking and cycling. For example, Flint et al. used a representative sample of the population of the United Kingdom to investigate associations of commute mode with Body Mass Index (BMI) and body fat [6]. This study identified that the adiposity benefits observed for those using public transport were similar to those walking or cycling to work. These benefits are hypothesised to be due to the fact that the use of public transport necessitates some physical activity to access interchange points – in the US for example, adults achieve a mean of 19 min a day of physical activity in the use of public transport to commute to work [7].

Physical activity is particularly important for those at older ages, being linked to healthy ageing and maintaining good physical functioning [8, 9] . Nonetheless, older adults are less likely to achieve recommended levels of physical activity [10]. Declines in work and leisure physical activity during later life mean that active transport may form a significant proportion of physical activity among older age groups [11–13]. Older age is additionally a time of significant life changes, including retirement, changes in income and residential moves all of which have been linked to changes in travel behaviour, including increases in public transport use [14–16]. In addition to this, recent policy changes in England have encouraged public transport use among an older population: since 2006 adults over the age of 60 years in England have been provided with a free bus pass in England, allowing cost-free travel on local buses (at any time within London and during off-peak times elsewhere in England). Previous work has linked the bus pass to increased walking and lower adiposity but has been limited in the strength of their conclusions because the available data resulted in reliance on analyses of cross-sectional associations between the bus pass and walking [12] and on a measure of eligibility for a bus pass, rather than holdership [17].

Interventional studies among working adults have linked changes from inactive to active forms of travel to lower BMI [18], a finding supported using longitudinal observational data from UK Biobank [15, 19]. However, the majority of studies examining associations between public transport use and health are based on cross-sectional analyses, and further longitudinal research is needed [20]. Public transport may play a particularly important role for older people, for whom walking or cycling entire journeys may not be possible. Therefore, in light of national policies encouraging increased use of public transport in England, we have assessed the socio-demographic characteristics of people increasing their public transport use, and assessed whether these increases are related to adiposity and physical activity.

## Methods

### Sample and data

We used data from the English Longitudinal Study of Ageing (ELSA), a nationally representative cohort study of persons aged 50 years and older in England [21]. Data for this paper come from two waves of the ELSA sample: 2008 (wave 4; t0) that serves as baseline for the analyses (t0) and 2012 (wave 6; t1) which provides the follow-up data for the study (t1). These waves were chosen as the most recent data where participants were visited by a nurse to collect objective measures of adiposity.

We focus here on increases in public transport use rather than changes in use generally. This is due to policies such as the free bus pass potentially driving increases in public transport use as well as concerns that any decreases in public transport use would be the result of natural disease processes or ageing, and that analyses may not be able to be adequately control for these factors. Two separate analytical samples were created to assess different aspects of increases in public transport use. First, to assess predictors of increases in public transport we analysed individuals with complete data on public transport use at waves 4 and 6 who had increased their monthly use of public transport (sample *N* = 4834). Individuals who reported the same level of public transport use at waves 4 and 6 were used as the reference group, while those who decreased their public transport use were excluded. Participants with the highest level of use at baseline were excluded due to being unable to increase their frequency of use (*N* = 364) Second, to assess initiating public transport use we further restricted the sample described above to include only individuals who reported not using public transport at baseline (*N* = 2064). A full breakdown of the levels of public transport use at baseline and follow up is presented in Appendix Table 6.

### Variables

Public transport use was assessed with the question “How often do you use public transport?” with six potential responses: every day or nearly every day; two or three times a week; once a week; two or three times a month; once a month or less; and Adiposity was objectively measured as part of the ELSA nurse visits. BMI was calculated from height and weight measured using, respectively, a stadiometer and calibrated Tanita-305 scales. Waist circumference was measured using a popper fixing tape measure. Physical activity was derived from three questions on frequency participants undertook mild, moderate and vigorous activities. Possible answers were: more than once a week; once a week; 1 to 3 times a month; hardly ever; or never. In line with previous work we categorised those partaking in vigorous activities at least once a month, or moderate activities at least once a week, as undertaking at least some physical activity [22].

We adjusted our models for a range of factors known, from the established literature [11, 14–16, 18, 23], to be linked to either or both of public transport use and our outcomes. In analyses of increases in public transport use these are included as potential predictors of these increases, while in models of our health outcomes we conceptualised these factors as potential confounders of our relationships of interest. The majority of these were constructed to account for variations between baseline and follow up. These were age; quadratic age; gender; non-pension household wealth quintile at baseline and changes in this; participation in paid work (in paid work at baseline and follow up; retiring between baseline and follow up; retired at both waves); changes in physical activity; household access to a car at baseline and follow up; and residential relocation between baseline and follow up.

Eligibility for a free bus pass was assessed based on age of participants at interview. Between the two time points in this study eligibility changed in line with the increasing state pension age for women, such that in 2008 all adults ≥60 years were eligible, but this was 61 or 62 years by 2012, depending on exact date of birth. For confidentiality reasons, exact dates of birth are not included in the ELSA data and so we categorised only those aged ≥62 years when interviewed as eligible in 2012 [17].

We also created variables measuring changes in problems with Activities of Daily Living (ADL), and mobility problems. ADLs are indicators of the functional status of older adults [24] and in ELSA participants were asked if they had problems with six tasks such as eating or getting in and out of bed. Mobility problems have previously been associated with both reduced use of public transport and with our health markers [17]. Participants were asked about problems with 10 tasks such as sitting for two hours or picking up a coin [21]. We categorised participants as having none, or one or more of these mobility problems, and assessed changes between baseline and follow up.

### Statistical analyses

The two analytic samples were analysed separately for analyses on increases in public transport analysis and the initiating public transport respectively. Descriptive characteristics were first described as frequencies for categorical variables or mean values for continuous variables. Logistic regression was used to assess predictors of initiating, and increasing, public transport use between baseline and follow up. Analyses of both initiating and increasing public transport use were adjusted for the covariates listed above, with the exception of adiposity models, which were not adjusted for changes in physical activity, which was considered to be on the causal pathway. Analyses of increases in public transport use were additionally adjusted for frequency of public transport use at baseline.

We identified the presence of statistically significant gender interactions for increases in public transport use with physical activity, BMI and waist circumference (all *p* < 0.001). These models were subsequently stratified by gender. Logistic (physical activity) and linear (BMI and waist circumference) regression models assessed the relationships with outcomes at follow-up, both unadjusted and adjusted for the potential confounders above. Outcomes at baseline were then added to these models to examine associations with changes over time. To examine whether any associations were mediated by non-transport physical activity we performed sensitivity analyses of the association between public transport use and adiposity, additionally adjusted for changes in physical activity levels.

All analyses were adjusted for survey weights provided by ELSA to account for non-response among certain groups.

## Results

Descriptive statistics for both samples are shown in Table 1. There were 4834 participants in the increasing public transport sample. Mean age in 2008 was 64.8 years and 46.9% were men. 67.7% were eligible for a free bus pass at both time points, while 9.1% became eligible. 42.3% never used public transport at baseline, 33.0% used public transport once a month or less, while 10.9% used public transport 2–3 times a week. 50.2% were retired at both time points, while 18.4% retired between baseline and follow up. 41.9% had mobility problems at both time points, and 12.4% developed mobility problems between baseline and follow up. 7.4% moved house between baseline and follow up.

**Table 1:** Description of the study population, England 2008–2012

	Increasing PT (% of total sample)	Initiating PT (% of total sample)
Overall N^a^	4834	2064
Mean age at t0 (SD)	64.8 (8.7)	65.0 (9.6)
Men	46.9	52.5
Women	53.1	47.5
Never eligible for bus pass	23.3	26.7
Became eligible	9.1	9.4
Always eligible	67.7	63.9
Never used PT at t0	42.3	100
Used PT once/month or less at t0	33.0	0
Used PT 2–3/month at t0	7.2	0
Used PT once/week at t0	6.5	0
Used PT 2–3/week at t0	10.9	0
Middle wealth group t0	20.6	22.1
Lowest wealth group t0	14.2	16.5
Second wealth group t0	18.3	21.1
Fourth wealth group t0	22.7	20.8
Highest wealth group t0	24.1	19.5
Stable wealth t0 to t1	65.6	63.3
Wealth increase t0 to t1	16.5	17.4
Wealth decrease t0 to t1	17.9	19.4
Working t0 and t1	31.4	35.6
Retired t0 to t1	18.4	18.9
Retired t0 and t1	50.2	45.4
Physically inactive t0 and t1	11.3	17.8
Took up physical activity t0 to t1	7.5	7.6
Stopped physical activity t0 to t1	10.4	12.1
At least some physical activity t0 & t1	70.8	62.5
Access to a car t0 and t1	85.9	88.5
Lost access to car t0 to t1	5.1	5.0
Gained access to car t0 to t1	1.7	1.8
No access to a car t0 to t1	7.2	4.7
No ADLs t0 to t1	76.5	70.1
Developed ADLs t0 to t1	7.9	9.8
Recovered from ADL t0 to t1	6.0	6.1
ADLs t0 and t1	9.6	14.0
No mobility problems t0 to t1	35.6	31.4
Developed mobility problems t0 to t1	12.4	13.2
Improved mobility problems t0 to t1	10.1	9.2
Mobility problems both t0 and t1	41.9	46.3
Same address t0 to t1	92.6	91.7
Moved address t0 to t1	7.4	8.3

*SD* Standard Deviation, *ADL* Activities of Daily Living

^a^This total N includes respondents with the same level of transport at both time points

There were 2064 participants in the initiating public transport sample. The mean age in 2008 was 65.0 years and 52.5% were men. 63.9% were eligible for a free bus pass at both time points and 9.4% became eligible for a bus pass. 45.4% were retired at both time points and 18.9% retired during the study period. 46.3% had mobility problems at both time points, 13.2% developed mobility problems during the study, and 8.3% moved house between baseline and follow up.

Table 2 displays summary statistics and results from logistic regression among those who increased their public transport use. 33.0% of the sample increased their use of public transport between baseline and follow up. Participants becoming eligible for a bus pass were more likely to increase public transport use compared with those never eligible (47.6% vs. 31.6%). These differences persisted in fully adjusted models – AOR 1.77, 95% CI 1.35; 2.33. Those using public transport between twice a month and once a week at baseline were also more likely to increase their use compared with those never using public transport at baseline. For example, 49.2% of those using public transport once a week at baseline increased their public transport use compared with 32.5% among never users (AOR 1.70, 1.32; 2.19). People retiring during the study period were more likely to increase public transport use than those working at both time points (40.7% vs. 31.3%, AOR 1.57, 1.29; 1.91). Participants who moved house were more likely to increase public transport use than those who did not (39.6% vs. 32.5%, AOR 1.37, 1.08; 1.72).

**Table 2:** Results from logistic regression predicting increase in public transport use between 2008 (t0) and 2012 (t1), England

	% of analytic sample who increased PT use (t0 – t1)	AOR	95% CI
Overall (*N* = 4834)	33.0	–	–
Age in years	–	0.98	0.96; 1.00
Quadratic age	–	1.00	1.00; 1.00
Men	32.7	ref	ref
Women	33.2	1.03	0.90; 1.17
Never eligible for bus pass	31.6	ref	ref
Became eligible	47.6	1.77	1.35; 2.33
Always eligible	31.5	1.06	0.76; 1.48
Never used PT at t0	32.4	ref	ref
Used PT once/month or less at t0	30.4	0.77	0.67; 0.90
Used PT 2–3/month at t0	48.9	1.70	1.34; 2.16
Used PT once/week at t0	49.2	1.70	1.32; 2.19
Used PT 2–3/week at t0	23.1	0.48	0.37; 0.61
Middle wealth group t0	33.2	ref	ref
Lowest wealth group t0	32.6	1.03	0.81; 1.30
Second wealth group t0	31.9	0.93	0.75; 1.16
Fourth wealth group t0	33.1	0.92	0.75; 1.12
Highest wealth group t0	33.8	0.92	0.74; 1.13
Stable wealth t0 to t1	32.6	ref	ref
Wealth increase t0 to t1	32.7	0.95	0.79; 1.15
Wealth decrease t0 to t1	34.8	1.14	0.96; 1.35
Working t0 and t1	31.3	ref	ref
Retired t0 to t1	40.7	1.57	1.29; 1.91
Retired t0 and t1	31.3	1.37	1.12; 1.69
Physically inactive t0 and t1	21.2	ref	ref
Took up physical activity t0 to t1	34.8	1.80	1.29; 2.49
Stopped physical activity t0 to t1	28.7	1.45	1.07; 1.97
At least some physical activity t0 & t1	35.3	1.80	1.38; 2.35
Access to a car t0 and t1	32.5	ref	ref
Lost access to car t0 to t1	49.6	3.71	2.74; 5.01
Gained access to car t0 to t1	26.2	1.08	0.64; 1.82
No access to a car t0 to t1	28.9	1.57	1.17; 2.11
No ADLs t0 to t1	35.5	ref	ref
Developed ADLs t0 to t1	27.2	0.73	0.58; 0.93
Recovered from ADL t0 to t1	30.9	1.05	0.82; 1.35
ADLs t0 and t1	19.4	0.64	0.51; 0.81
No mobility problems t0 to t1	36.0	ref	ref
Developed mobility problems t0 to t1	33.2	0.94	0.76; 1.16
Improved mobility problems t0 to t1	33.5	0.90	0.72; 1.13
Mobility problems both t0 and t1	30.3	1.00	0.84; 1.20
Same address t0 to t1	32.5	ref	ref
Moved address t0 to t1	39.6	1.37	1.08; 1.72

*PT* Public Transport, *AOR* Adjusted Odds Ratio, *CI* Confidence Intervals, *ADL* Activities of Daily Living

Table 3 displays summary statistics and results from logistic regression for participants who have initiated public transport use. 32.4% of the sample initiated public transport between baseline and follow up. Levels of initiating public transport use were higher among those becoming eligible for a free bus pass compared to those never eligible (50.5% vs. 34.4%, AOR 2.08, 1.37; 3.16) and among those retiring between baseline and follow up compared with those working at both time points (40.1% vs. 32.9%, AOR 1.57, 1.17; 2.11). Participants who moved house were also more likely to initiate public transport use (37.3% vs. 31.9%, AOR 1.53, 1.08; 2.19).

**Table 3:** Results from logistic regression predicting initiation of public transport use between 2008 (t0) and 2012 (t1), England

	% of analytic sample who initiated PT use (t0 – t1)	AOR	95% CI
Overall (*N* = 2064)	32.4	–	–
Age in years	–	0.96	0.93; 0.98
Quadratic age	–	1.00	1.00; 1.00
Men	32.9	ref	ref
Women	31.8	1.14	0.93; 1.40
Never eligible for bus pass	34.4	ref	ref
Became eligible	50.5	2.08	1.37; 3.16
Always eligible	28.9	1.30	0.78; 2.17
Middle wealth group t0	34.7	ref	ref
Lowest wealth group t0	23.4	0.80	0.56; 1.15
Second wealth group t0	28.8	0.79	0.57; 1.10
Fourth wealth group t0	33.6	0.82	0.61; 1.12
Highest wealth group t0	39.8	1.03	0.74; 1.42
Stable wealth t0 to t1	31.1	ref	ref
Wealth increase t0 to t1	31.8	0.92	0.69; 1.22
Wealth decrease t0 to t1	37.1	1.29	0.99; 1.67
Working t0 and t1	32.9	ref	ref
Retired t0 to t1	40.1	1.57	1.17; 2.11
Retired t0 and t1	28.7	1.63	1.18; 2.24
Physically inactive t0 and t1	12.6	ref	ref
Took up physical activity t0 to t1	31.4	2.15	1.30; 3.54
Stopped physical activity t0 to t1	20.6	1.37	0.86; 2.19
At least some physical activity t0 & t1	40.4	2.84	1.89; 4.27
Access to a car t0 and t1	34.2	ref	ref
Lost access to car t0 to t1	28.4	2.12	1.25; 3.59
Gained access to car t0 to t1	13.5	0.73	0.26; 2.04
No access to a car t0 to t1	10.3	0.74	0.35; 1.54
No ADLs t0 to t1	37.2	ref	ref
Developed ADLs t0 to t1	26.0	1.02	0.70; 1.5
Recovered from ADL t0 to t1	24.2	0.84	0.52; 1.35
ADLs t0 and t1	16.0	0.80	0.53; 1.20
No mobility problems t0 to t1	41.6	ref	ref
Developed mobility problems t0 to t1	34.9	0.85	0.62; 1.16
Improved mobility problems t0 to t1	36.7	0.89	0.63; 1.27
Mobility problems both t0 and t1	24.5	0.77	0.59; 1.02
Same address t0 to t1	31.9	ref	ref
Moved address t0 to t1	37.3	1.53	1.08; 2.19

*AOR* Adjusted Odds Ratio, *CI* Confidence Intervals, *ADL* Activities of Daily Living

Associations of increasing levels of public transport use and health markers are shown in Table 4. Among both men and women, increasing public transport use was associated with an increased likelihood of undertaking at least some physical activity at follow up (e.g. AOR 1.75, 1.04; 2.96 among men). For women this association was also evident after adjusting for baseline physical activity (AOR = 1.67, 1.03; 2.72). Women who increased public transport use had mean BMI 2.03 kg/m^2^ lower (− 2.84; − 1.21) at follow up than those who did not, although this was attenuated after adjusting for BMI at baseline (− 0.40 kg/m^2^, − 0.82; 0.01). Results for waist circumference were similar to BMI findings, although not statistically significant for men.

**Table 4:** Associations of increasing public transport use and physical activity / adiposity, 2008/09–2012/13 England

	Model 1: Unadjusted associations at t1 (CI)	Model 2: Fully adjusted at t1 (CI)	Model 3: Unadjusted change in outcome t0 to t1 (CI)	Model 4: Fully adjusted change in outcome t0 to t1 (CI)
Physical Activity^a^ (Adjusted Odds Ratios)				
Men	**2.52 (1.65; 3.84)**	**1.75 (1.04; 2.96)**	**2.11 (1.32; 3.37)**	1.69 (0.99; 2.88)
Women	**4.06 (2.78; 5.92)**	**2.03 (1.27; 3.23)**	**2.61 (1.70; 4.01)**	**1.67 (1.03; 2.72)**
Body Mass Index (Coefficients in kg/m^2^)				
Men	**−0.76 (−1.39; − 0.13)**	−0.50 (− 1.09; 0.09)	−0.02 (− 0.26; 0.21)	−0.01 (− 0.23; 0.21)
Women	**−2.30 (− 3.22; − 1.39)**	**−2.03 (− 2.84; − 1.21)**	− 0.33 (− 0.74; 0.09)	− 0.40 (− 0.82; 0.01)
Waist Circumference (Coefficients in centimetres)				
Men	**−2.58 (−4.29; − 0.87)**	−1.39 (− 2.99; 0.22)	− 0.74 (− 1.57; 0.10)	− 0.62 (− 1.45; 0.21)
Women	**− 5.14 (− 7.16; − 3.11)**	**−4.11 (− 5.98; − 2.25)**	− 0.93 (− 2.08; 0.22)	−1.10 (− 2.26; 0.05)

t0 = 2008/09 t1 = 2012/13

Models adjusted for age (years); quadratic age; wealth group at t0; changes in wealth group t0 to t1; working status t0 to t1; household access to a car at t0 and t1; problems with Activities of Daily Living at t0 and t1; problems with mobility at t0 and t1; moving house between t0 and t1

Model 1: Unadjusted cross-sectional associations at follow up (t1; 2012/13)

Model 2: Fully adjusted cross-sectional associations at follow up (t1; 2012/13)

Model 3: Unadjusted cross-sectional associations at follow up (t1; 2012/13), controlled for baseline (t0; 2008/09) outcome

Model 4: Fully adjusted cross-sectional associations at follow up (t1; 2012/13), controlled for baseline (t0; 2008/09) outcome

*CI* Confidence Intervals

^a^Physical activity defined as undertaking moderate activity at least once a week, or vigorous at least once a month

bold denotes statistical significance at *p*< = 0.05

Associations of initiating public transport use with health markers are shown in Table 5. Initiating public transport use was associated with an increased likelihood of undertaking at least some physical activity at follow up for both men and women (e.g. AOR for men 1.78, 1.07; 2.97). These associations were not substantially attenuated by adjustments for physical activity at baseline (e.g. AOR for men 1.70, 1.01; 2.86). Women who took up public transport between baseline and follow up had lower BMI at follow up (− 1.95 kg/m^2^, − 2.75; − 1.15), although this association was not statistically significant after adjustment for BMI at baseline (− 0.38 kg/m^2^,-0.77; 0.01). Results for waist circumference were similar to BMI results for both genders and associations were not statistically significant for men.

**Table 5:** Associations of initiating public transport and physical activity / adiposity, 2008–2012 England

	Model 1: Unadjusted associations at t1 (CI)	Model 2: Fully adjusted at t1 (CI)	Model 3: Unadjusted change in outcome t0 to t1 (CI)	Model 4: Fully adjusted change in outcome t0 to t1 (CI)
Physical Activity^a^ (Odds Ratios)				
Men	**2.54 (1.70; 3.79)**	**1.78 (1.07; 2.97)**	**2.11 (1.36; 3.27)**	**1.70 (1.01; 2.86)**
Women	**3.70 (2.62; 5.23)**	**1.95 (1.24; 3.08)**	**2.23 (1.50; 3.33)**	**1.61 (1.00; 2.60)**
Body Mass Index (Coefficients in kg/m^2^)				
Men	**−0.71 (− 1.31; − 0.11)**	−0.50 (− 1.09; 0.08)	0.02 (− 0.21; 0.25)	0.02 (− 0.20; 0.24)
Women	**− 2.04 (− 2.89; − 1.20)**	**−1.95 (− 2.75; − 1.15)**	− 0.24 (− 0.60; 0.13)	−0.38 (− 0.77; 0.01)
Waist Circumference (Coefficients in centimetres)				
Men	**−2.36 (− 3.99; − 0.73)**	−1.26 (− 2.83; 0.31)	−0.59 (− 1.39; 0.21)	−0.57 (− 1.40; 0.25)
Women	**−4.72 (− 6.66; − 2.78)**	**− 3.86 (− 5.73; − 2.00)**	− 0.88 (− 1.96; 0.20)	−1.04 (− 2.18; 0.10)

t0 = 2008/09 t1 = 2012/13

Model 1: Unadjusted cross-sectional associations at follow up (t1; 2012/13)

Model 2: Fully adjusted cross-sectional associations at follow up (t1; 2012/13)

Model 3: Unadjusted cross-sectional associations at follow up (t1; 2012/13), controlled for baseline (t0; 2008/09) outcome

Model 4: Fully adjusted cross-sectional associations at follow up (t1; 2012/13), controlled for baseline (t0; 2008/09) outcome

Fully adjusted models adjusted for: age (years); quadratic age; wealth group at t0; changes in wealth group t0 to t1; working status t0 to t1; household access to a car at t0 and t1; problems with Activities of Daily Living at t0 and t1; problems with mobility at t0 and t1; moving house between t0 and t1

*CI* Confidence Intervals

^a^Physical activity defined as undertaking moderate activity at least once a week, or vigorous at least once a month

bold denotes statistical significance at *p*< = 0.05

Results from sensitivity analyses of adiposity additionally adjusted for physical activity were similar (Appendix Tables 7 and 8). In analyses not adjusted for baseline adiposity women had lower levels of adiposity at follow up, although these relationships were attenuated by adjustments for baseline adiposity.

## Discussion

This longitudinal analysis of a representative sample of older adults living in England links increases in public transport use with increases in physical activity levels and lower adiposity, although the latter finding was attenuated in models which adjusted for baseline adiposity. Older adults were more likely to both initiate public transport and to increase their levels of use of public transport when they became eligible for the national free bus pass scheme. Retirement and moving house were also linked to both initiating and increasing public transport use.

### Comparison with other studies

Retiring from paid work and moving house emerged here as significant predictors of changes in public transport use. A previous longitudinal analysis of UK Biobank among adults aged 40–69 years found that changes in household income were associated with variations in active travel [15]. Changes in income may be indicative of retirement or job changes, the latter which in turn affect the start and end points of journeys, in common with house moves [16]. Changes in public transport use in our sample were considerably higher than identified among younger respondents in work on commute mode changes (e.g. 32% of older adults took up public transport here compared with 15% of adults changing commute mode [15]). These differences may be in part due to different study designs and follow up times, but nonetheless the high level of increases in public transport identified here suggest that retiring from paid work may be an opportune time to intervene in the life course to change travel behaviours and physical activity, in line with other work on behaviour changes around retirement [25].

Previous work has investigated the impact of the free bus pass scheme in England. Analyses of 2004–2008 National Travel Survey data found the introduction of the pass to be linked to more public transport use and walking, with effects equitable across markers of socio-economic position [12]. Analyses of 2002–2008 ELSA data found that those using public transport in 2008 were less likely to have become obese, although these relationships were evident for BMI-measured obesity but not for waist circumference [26]. We did not find any relationships between wealth and increases in public transport use, which may be due to the existence of the free bus pass with high levels of uptake ameliorating potentially inequitable effects [17]. The current study extends previous work using more granular data on public transport use to examine both initiating and increasing public transport use alongside detailed data on changes in life circumstances.

While this is the first study to our knowledge to examine increases in public transport use alongside adiposity and physical activity measures among older adults, previous work among working age adults has linked changes from private motorised to public transport with reduced BMI [15, 18, 19]. Cross-sectional work has found older adults with a free bus pass to be more likely to be physically active than those without [17]. Increases in physical activity, even late in life, have been associated with a variety of health outcomes including major chronic disease, depressive symptoms, cognitive impairment and mortality [8, 27].

There are strengths and limitations to this study. ELSA provides a large and representative sample of older adults in England, and the longitudinal nature allowed us to specifically investigate predictors of changes and their effects (using time-varying predictors and outcomes). The adiposity measures used were objective, and the use of both BMI and waist circumference strengthens our confidence in these findings. There are nevertheless some limitations. We have followed participants up over four years: however, we do not know at which point in these four years they have increased their public transport use. Also, assessment of public transport use and physical activity were both self-reported, although the physical activity measure has demonstrated excellent convergent validity with a variety of risk factors in previous work [27–29]. Changes in the public transport use questions in ELSA meant that it was not possible to examine these issues over a longer period using earlier waves.

### Policy implications

This study highlights the potential role of public transport in increasing population levels of physical activity. The possibility of incorporating more walking or cycling into public transport journeys is noted in National Institute for Health and Clinical Excellence (NICE) guidance on walking and cycling [30], in part as this represents an option among people who are unwilling or unable to walk or cycle entire journeys. As changes in public transport use identified here were considerably more common than changes to walking and cycling identified in other research, this represents an important opportunity to increase population levels of physical activity through active travel. The potential health benefits of active travel are additionally most pronounced among those who are otherwise physically inactive [31, 32].

The national free bus pass scheme is a universal benefit linked to the state pension age for women, and has been demonstrated here to increase public transport use among those becoming eligible. The scheme costs in the region of £1billion a year [33], but the use of public transport among this age group supports local economies through spending in shops as well as voluntary work and childcare, meaning that net costs of the scheme are likely overstated [34]. Calls for means-testing of this scheme are undermined since, at a time of life where all groups are at risk or obesity and physical inactivity, the universal nature of the bus pass provides an important mechanism to improve health across the whole population rather than benefitting only the most affluent in society [23].

## Conclusion

The key predictors of both initiating and increasing public transport among older adults were becoming eligible for a bus pass, retirement and moving house. Both initiating and increasing public transport use were associated with increased physical activity and may be associated with lower adiposity. These findings strengthen the case for considering public transport provision as an effective means of promoting healthier ageing.

## Appendix

**Table 6:** levels of public transport use at 2008/09 (t0) and 2012/13 (t1)

	Never used PT at t1	Used PT once/month or less at t1	Used PT 2–3/month at t1	Used PT once/week at t1	Used PT 2–3/week at t1	Used PT every day or nearly every day at t1	Total
Never used PT at t0 (N)	1393	494	73	42	34	28	2064
%	67.49	23.93	3.54	2.03	1.65	1.36	100
Used PT once/month or less at t0 (N)	490	1124	272	97	100	23	2106
%	23.27	53.37	12.92	4.61	4.75	1.09	100
Used PT 2–3/month at t0 (N)	79	243	180	90	73	13	678
%	11.65	35.84	26.55	13.27	10.77	1.92	100
Used PT once/week at t0 (N)	40	88	108	160	137	22	555
%	7.21	15.86	19.46	28.83	24.68	3.96	100
Used PT 2–3/week at t0 (N)	54	64	83	130	408	122	861
%	6.27	7.43	9.64	15.1	47.39	14.17	100
Used PT every day or nearly every day at t0 (N)	28	25	13	28	144	291	529
%	5.29	4.73	2.46	5.29	27.22	55.01	100

**Table 7:** Associations of increased public transport use and adiposity, after adjusting for physical activity

	Model 1: Unadjusted associations at t1 (CI)	Model 2: Fully Aadjusted at t1 (CI)	Model 3: Unadjusted change in outcome t0 to t1 (CI)	Model 4: Fully Aadjusted change in outcome t0 to t1 (CI)
Body Mass Index (Coefficients in kg/m^2^)				
Men	**−0.76 (1.39; −0.13)**	−0.50 (−1.09; 0.10)	−0.02 (−0.26; 0.21)	−0.01 (− 0.24; 0.21)
Women	**−2.58 (−4.29; − 0.84)**	−1.25 (−2.86; 0.37)	−0.74 (− 1.57; 0.10)	−0.57 (− 1.42; 0.27)
Waist Circumference (Coefficients in centimetres)				
Men	**−2.30 (−3.22; − 1.39)**	**−1.73 (− 2.52; − 0.93)**	−0.33 (− 0.74; 0.09)	−0.37 (− 0.79; 0.05)
Women	**− 5.14 (− 7.16; − 3.11)**	**−3.43 (− 5.28; − 1.58)**	−0.93 (− 2.08; 0.22)	−1.08 (− 2.25; 0.08)

t0 = 2008/09 t1 = 2012/13

Models adjusted for age (years); quadratic age; wealth group at t0; changes in wealth group t0 to t1; working status t0 to t1; household access to a car at t0 and t1; problems with Activities of Daily Living at t0 and t1; problems with mobility at t0 and t1; moving house between t0 and t1; and physical activity between t0 and t1

Model 1: Unadjusted cross-sectional associations at follow up (t1; 2012/13)

Model 2: Fully adjusted cross-sectional associations at follow up (t1; 2012/13)

Model 3: Unadjusted cross-sectional associations at follow up (t1; 2012/13), controlled for baseline (t0; 2008/09) outcome

Model 4: Fully adjusted cross-sectional associations at follow up (t1; 2012/13), controlled for baseline (t0; 2008/09) outcome

*CI* Confidence Intervals

bold denotes statistical significance at *p*< = 0.05

**Table 8:** Associations of initiating public transport and adiposity, after adjusting for physical activity

	Unadjusted at t1 (CI)	Adjusted at t1 (CI)	Unadjusted change t0 to t1 (CI)	Adjusted change t0 to t1 (CI)
Body Mass Index (Coefficients in kg/m^2^)				
Men	**−0.71 (−1.31; − 0.11)**	−0.50 (− 1.09; 0.08)	0.02 (− 0.21; 0.25)	0.01 (− 0.21; 0.23)
Women	**−2.04 (− 2.89; − 1.20)**	**−1.67 (− 2.44; − 0.90)**	−0.24 (− 0.60; 0.13)	−0.37 (− 0.76; 0.02)
Waist Circumference (Coefficients in centimetres)				
Men	**−2.36 (−3.99; − 0.73)**	−1.26 (− 2.83; 0.31)	−0.59 (− 1.39; 0.21)	−0.57 (− 1.40; 0.25)
Women	**−4.72 (− 6.66; − 2.78)**	**−3.18 (− 5.02; − 1.35)**	−0.88 (− 1.96; 0.20)	−1.01 (− 2.15; 0.13)

t0 = 2008/09 t1 = 2012/13

Models adjusted for age (years); quadratic age; wealth group at t0; changes in wealth group t0 to t1; working status t0 to t1; household access to a car at t0 and t1; problems with Activities of Daily Living at t0 and t1; problems with mobility at t0 and t1; moving house between t0 and t1; and physical activity between t0 and t1

Model 1: Unadjusted cross-sectional associations at follow up (t1; 2012/13)

Model 2: Fully adjusted cross-sectional associations at follow up (t1; 2012/13)

Model 3: Unadjusted cross-sectional associations at follow up (t1; 2012/13), controlled for baseline (t0; 2008/09) outcome

Model 4: Fully adjusted cross-sectional associations at follow up (t1; 2012/13), controlled for baseline (t0; 2008/09) outcome

*CI* Confidence Intervals

bold denotes statistical significance at *p*< = 0.05
